# Assessing Cannabidiol as a Therapeutic Agent for Preventing and Alleviating Alzheimer’s Disease Neurodegeneration

**DOI:** 10.3390/cells12232672

**Published:** 2023-11-21

**Authors:** Long Chen, Yuan Sun, Jinran Li, Sai Liu, Hancheng Ding, Guangji Wang, Xinuo Li

**Affiliations:** 1 Jiangsu Provincial Key Laboratory of Drug Metabolism and Pharmacokinetics, China Pharmaceutical University, 639 Longmian Avenue, Nanjing 211166, China; 2 State Key Laboratory of Natural Medicines, China Pharmaceutical University, 639 Longmian Avenue, Nanjing 211166, China

**Keywords:** Alzheimer’s disease, cannabidiol, microglia, astrocyte, neuroprotection, anti-inflammatory

## Abstract

Alzheimer’s disease (AD) is a leading neurodegenerative condition causing cognitive and memory decline. With small-molecule drugs targeting Aβ proving ineffective, alternative targets are urgently needed. Neuroinflammation, which is central to AD’s pathology, results in synaptic and neuronal damage, highlighting the importance of addressing inflammation and conserving neuronal integrity. Cannabidiol (CBD), derived from cannabis, is noted for its neuroprotective and anti-inflammatory properties, having shown efficacy in neuropathic pain management for epilepsy. To investigate the therapeutic efficacy of CBD in AD and to elucidate its underlying mechanisms, we aimed to contribute valuable insights for incorporating AD prevention recommendations into future CBD nutritional guidelines. Aβ_1–42_ was employed for in vivo or in vitro model establishment, CBD treatment was utilized to assess the therapeutic efficacy of CBD, and RNA-seq analysis was conducted to elucidate the underlying therapeutic mechanism. CBD mitigates Aβ-induced cognitive deficits by modulating microglial activity, promoting neurotrophic factor release, and regulating inflammatory genes. The administration of CBD demonstrated a protective effect against Aβ toxicity both in vitro and in vivo, along with an amelioration of cognitive impairment in mice. These findings support the potential inclusion of CBD in future nutritional guidelines for Alzheimer’s disease prevention.

## 1. Introduction

Alzheimer’s disease (AD) is the foremost neurodegenerative condition leading to senile dementia. Afflicted individuals primarily face memory degradation and diminished learning capacities [1]. The economic and emotional burdens of AD present profound challenges for families [2,3,4]. Despite rigorous research endeavors, no current therapeutic solution can fully mitigate or reverse AD’s clinical symptoms.

AD is a multifaceted, diverse, and progressive pathology, hallmarked by extracellular plaques rich in β-amyloid (Aβ), intracellular neurofibrillary tangles containing tau, neuronal degeneration, and neuroinflammation [1,5,6]. Yet, most of the drugs targeting Aβ in clinical trials have proven to be unsuccessful, underscoring the urgency of identifying efficacious and non-toxic treatments that operate on alternative molecular mechanisms.

Recent insights from neuroinflammation studies have suggested a coordinated role of microglia, astrocytes, and neurons in accelerating neurodegeneration. Specifically, Aβ is known to activate the nuclear factor-κB (NF-κB) pathway in astrocytes, which subsequently elevates the release of complementary C3. C3 then engages with C3a receptors on neurons and microglia, triggering neuronal impairment and microglial activation [7]. Conversely, stimulated microglia can provoke A1 neurotoxic astrocytes by discharging IL-1α, C1q, and TNF-α [8]. This dynamic interrelation between microglia and astrocytes potentially fosters a continuous inflammatory cycle within the AD environment. The ensuing secretion of pro-inflammatory molecules, notably IL-1β and TNF-α, can induce synaptic anomalies, neuronal loss, and hindered neurogenesis [9]. Additionally, the activation of the complement system might amplify the phagocytic function of the microglia, risking inappropriate synaptic elimination [10].

Compounds derived from the Cannabis sativa plant have historically been employed for various therapeutic purposes [11]. Of more than 80 phytocannabinoids in cannabis, CBD has attracted significant scientific interest due to its abundance and therapeutic potential [12]. Recognized as a non-addictive cannabinoid, the FDA’s endorsement of a CBD formulation for severe seizure disorders underscores its potential to modulate neural activity [13,14]. Prior studies have elucidated CBD’s efficacy in ameliorating multiple sclerosis (MS) symptoms by modulating autoimmune T cells and attenuating neuropathic pain through neuroinflammation inhibition [15,16]. Furthermore, CBD has been shown to down-regulate GSK-3β, a principal inhibitor of the Wnt/β-catenin pathway implicated in neuroinflammation regulation, offering therapeutic benefits in epilepsy [17]. Additionally, CBD’s neuroprotective attributes have been documented in experimental cerebral ischemia models [18].

The detection of heightened inflammatory markers in AD patients, coupled with the linkage between AD susceptibility genes and innate immune functionalities, has accentuated the pivotal role of inflammation in the etiology of AD [19,20,21]. A plethora of therapeutic strategies addressing neuroinflammation in AD have been investigated [19,22,23], predominantly focusing on modulation of the NF-κb, NLPR3-caspase1, and p38MAPK pathways. These strategies were designed to attenuate the proinflammatory microglial phenotype and mitigate the memory impairments observed in AD animal models. Given the established anti-inflammatory attributes of CBD within the neural milieu, its prospective therapeutic utility in AD has been hypothesized [24,25]. However, a comprehensive understanding of the exact mechanisms underpinning CBD’s anti-inflammatory actions in AD is still forthcoming. This research aims to deliver a meticulous evaluation of CBD’s therapeutic promise in AD, shedding light on its potential synergy with extant AD treatments and possibly introducing novel therapeutic approaches for the clinical oversight of AD.

## 2. Materials and Methods

### 2.1. Cell Culture and Cytotoxicity Test In Vitro

The SH-SY5Y cell line (Shanghai Zhongqiaoxinzhou Company, Shanghai, China) was cultured in Dulbecco’s modified Eagle medium (Invitrogen, Waltham, MA, USA), which was added to 10% FBS, 100 IU/mL penicillin, and 100 µg/mL streptomycin in a humidified incubator with 5% CO_2_ at 37 °C.

The SH-SY5Y cells were seeded into 96-well plates and incubated overnight until they reached approximately 70% confluence. Cells in the vehicle group were cultured in DMEM medium containing vehicle for 36 h, while those in the Aβ treatment group were cultured with DMEM medium for 12 h and then treated with 10 μM Aβ_1–42_ for an additional 24 h. In the CBD treatment group, cells were pretreated with CBD at concentrations of 0.25, 0.5, 2.5, and 5 μM for a duration of 12 h before being exposed to 10 μM Aβ_1–42_ for another period of 24 h. Finally, cell viabilities were assessed using the MTT method.

### 2.2. Animals and Drug Administration

All male C57/BL6 mice (8 weeks old, weight 20–22 g) were purchased from SPF (Beijing) Biotechnology Company (Beijing, China). The animal culture and experiments were conducted in accordance with the ethical guidelines of the Ministry of Science and Technology of the People’s Republic of China and approved by the Center for Pharmaceutical Laboratory Animals of China Pharmaceutical University.

Recombinant human Aβ_1–42_ peptide (Beyotime Biotechnology, Shanghai, China) was solubilized using sterile PBS for a final Aβ_1–42_ solution concentration of 2 mg/mL. Afterward, the Aβ_1–42_ solution was incubated at 37 °C for 24 h to obtain aggregated Aβ_1–42_. All mice were randomly divided into three groups (*n* = 6 per group): a sham+Veh (vehicle) group, an Aβ (intracerebroventricular injection, i.c.v.) + Veh group, and an Aβ+CBD (25 mg/kg, intragastric administration, i.g.) group. The sham+Veh group was injected with 5 μL PBS, and the other mice were treated with 5 μL aggregated Aβ_1–42_, which was inserted into the lateral ventricle using a brain stereo-positioning instrument (Harvard). CBD powder (PUSH BIO-TECHNOLOGY, Chengdu, China) was dissolved in a mixture of reagents (ethanol, Tween-80 and saline, 1:1:18), and the mice were given CBD by gavage continuously for 13 days after surgery. Mice in the AD + Veh and sham + Veh group were gavaged with normal saline.

### 2.3. Morris Water Maze

On the 8th day after operation, a Morris water maze was utilized for cognitive function measurement. The MWM system mainly consists of a black circular pool, an aqueous solution dyed white by titanium dioxide powder, a circular concealed platform 1 cm underwater, and a video analysis system. The experimental maze was divided into four quadrants. We systematically altered the platform’s position within the four quadrants on a daily basis, designating the quadrant where the platform was situated as the third. The experiment lasted for a total of 5 days, consisting of 4 consecutive days of hidden platform training followed by a probe trial on the 5th day. During the hidden platform training, mice were given a swimming time of 90 s to locate the target platform, and this was repeated four times. Each mouse entered the pool from a consistent starting position opposite the third quadrant. The video analysis system automatically recorded the time at which the mouse successfully climbed onto the platform. If a mouse could not reach the platform within 90 s, it was artificially led to the target and allowed to remain on the platform for 10 s to find its location. On day 5, a probe trial was conducted in which various parameters, such as latency, path length, swimming velocity, target quadrant residence time, and traveled trajectory, were recorded and analyzed using video tracking technology.

### 2.4. RNA Extraction and Real-Time PCR

The mice were sacrificed under deep anesthesia and perfused transcardially with frozen PBS for 2 min (PH = 7.4). Brain tissues were stored at −20 °C.

Total RNA was obtained from the cerebral cortex using Trizol Reagent (Vazyme, Nanjing, China) and reverse transcribed to cDNA with HiScript III RT SuperMix (Vazyme). The primer sequence is listed in Table 1. The real-time PCR was performed as described previously [26]. In brief, it was carried out via the CFX Opus Real-Time PCR System (Bio-Rad, Hercules, CA, USA) using Taq Pro Universal SYBR qPCR Master Mix (Vazyme). Relative expression changes were calculated using the 2^−ΔΔCt^ method, and the target gene value was normalized to GAPDH.

### 2.5. Immunofluorescence

Mice were intraperitoneally perfused with frozen PBS (pH = 7.4) for 1 min, followed by perfusion with 4% paraformaldehyde (PFA) for 1 min to fix the tissues. After cardiac perfusion, the brains were collected and fixed in 4% PFA at 4 °C for a minimum of 24 h under light protection. The fixed brain tissue was then dehydrated in a solution of 30% sucrose. After 48 h, the brain tissue sank to the bottom of the liquid and was cut into coronal sections that were 25 μm thick using a cryomicrotome (Leica, Wetzlar, Germany, CM1950). The intact hippocampal sections were stored in cryosolution containing PBS, ethylene glycol, and glycerol at a ratio of 5:3:2.

The brain slices were penetrated with 0.3% Triton X-100 (Beyotime Biotechnology, ST795) for 20 min. Following blocking with a solution of bovine serum albumin diluted to a concentration of 5% in PBS at room temperature for one hour, the slices were incubated overnight at 4 °C with primary antibodies (rabbit anti-iBA1 (Fujifilm, 019-19741; dilution: 1:300, Tokyo, Japan), rabbit anti-GFAP (CST, 80788; dilution:1:200)). Subsequently, the secondary antibody (Goat Anti-Rabbit IgG H&L (Alexa Fluor^®^488) (Abcam, ab150077; dilution: 1:500, Cambridge, UK)) was applied for one hour at room temperature, and, finally, the slices were stained with DAPI (Beyotime Biotechnology, C1002; dilution: 1 μg/mL) for ten minutes. After being washed several times in PBS, the samples were analyzed using fluorescence microscopy (BioTek, Cytation5, Winooski, VT, USA), and images were captured using MicroImaging System (BioTek, Cytation5). Finally, the acquired images were analyzed and quantified using ImageJ software(1.54d).

### 2.6. RNA-Seq Analysis

Isolated RNA was subsequently used for RNA-seq analysis. cDNA library construction and sequencing were performed by Novogene (Beijing, China). High-quality reads were aligned to the mouse reference genome using Hisat2 (v2.0.3). We identified DEGs between the samples and performed clustering analysis and functional annotation via R studio (v 4.2.3) and DESeq2 (v1.40.2). Trimmomatic (v 0.39) was used for quality control to assess the integrity of the analyzed RNA.

### 2.7. Statistical Analyses

The histograms and line charts were generated using GraphPad Prism 8.0 software. The results are presented as mean ± SEM (standard error of the mean). To ensure consistency, a minimum of three biological replicates were performed for all experiments. The unpaired Student’s *t*-test was employed to determine significant differences between groups. A one-way ANOVA and the original FDR method of Benjamini and Hochberg were used to compare multiple independent groups. Statistical significance is denoted by * when the *p*-value is < 0.05.

## 3. Results

### 3.1. CBD’s Neuroprotective Impact on Aβ-Induced Neuronal Cytotoxicity

**Figure 1:**
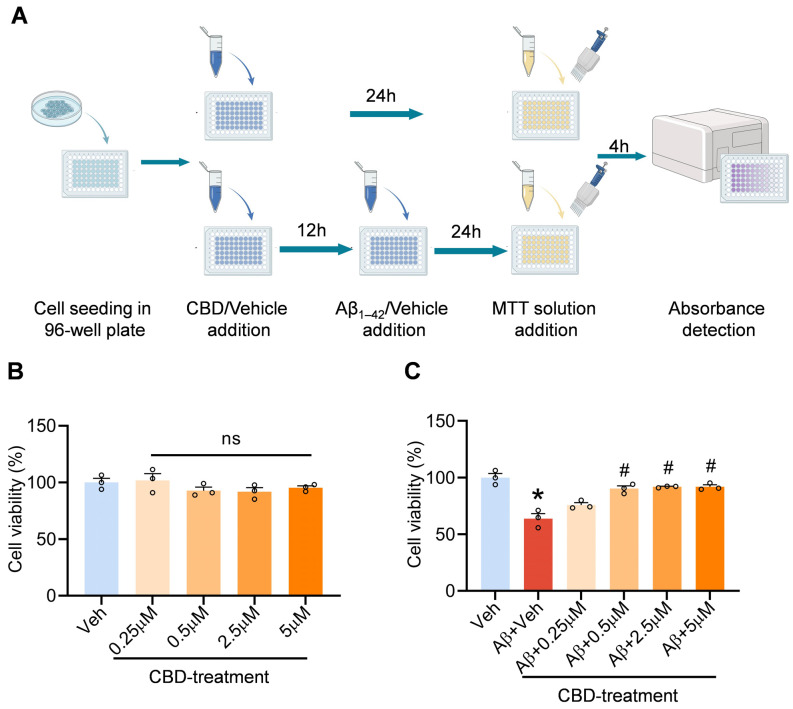
CBD mitigates Aβ-induced cytotoxicity in SH-SY5Y cells. (**A**) Experimental design schematic. (**B**) CBD cytotoxicity assessment in SH-SY5Y cells. (**C**) Effects of CBD on the cell viability of SH-SY5Y cells induced by Aβ_1–42_. Data are presented as mean ± SEM (*n* = 3). * *p* < 0.05 vs. sham + Veh group; ^#^ *p* < 0.05 vs. AD + Veh group; ns: not significant.

Memory deficits and cognitive disturbances are the cardinal clinical manifestations in AD patients, with neuronal loss being the primary pathological underpinning [1,27,28,29]. To elucidate the neuroprotective potential of CBD in neuronal substrates, we conducted cell viability assays. Prior to this, we assessed the cytotoxicity profile of CBD across a specific concentration spectrum. Our findings revealed an absence of notable cytotoxic effects of CBD on SH-SY5Y cells within the concentration range of 0.25 μM to 5 μΜ (Figure 1B). Concurrently, the MTT assay indicated a pronounced reduction in the viability of Aβ-stimulated SH-SY5Y cells, a decrement that CBD was capable of ameliorating. During the therapeutic evaluation across a CBD concentration gradient of 0.25 μM to 5 μM, CBD exhibited a dose-dependent therapeutic efficacy in the lower concentration spectrum, with a marked therapeutic impact observed at a concentration threshold of 0.5 μM. Collectively, these findings underscore CBD’s capacity to counteract the neuronal cytotoxicity instigated by Aβ_1–42_ (Figure 1C).

### 3.2. CBD’s Ameliorative Effects on Cognitive Deficits in Aβ_1–42_-Induced Mice

**Figure 2:**
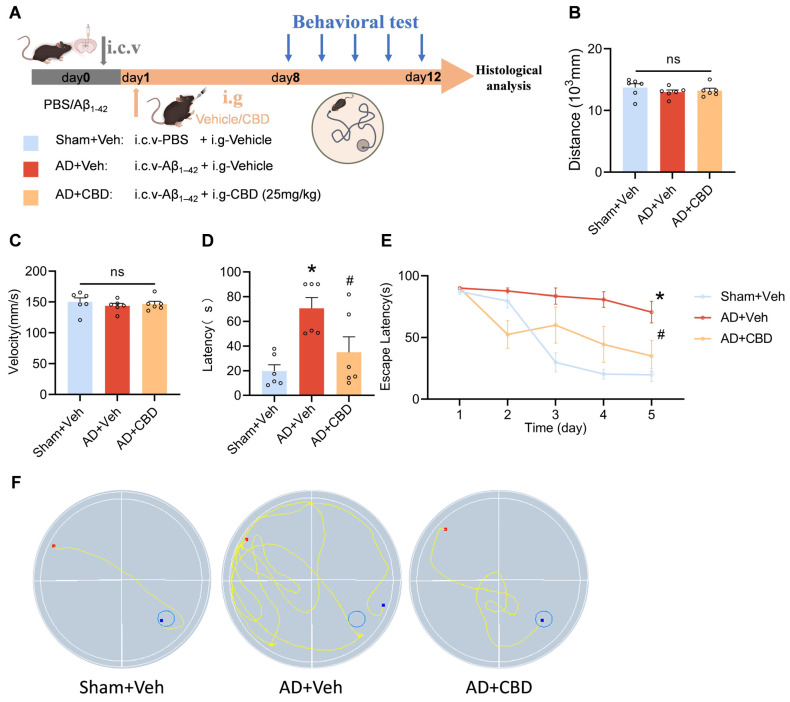
Behavioral and cognitive enhancement by CBD in Aβ_1–42_-induced mice. Mice were injected with Aβ_1–42_ (10 μg, 5 μL, i.c.v.), which was followed by CBD treatment (25 mg/kg, i.g.). (**A**) Experimental design schematic: (**B**) average swimming velocities across experimental groups; (**C**) traversed distances during the test by each group; (**D**) latency to locate the target platform during the final exploration trial; (**E**) temporal trend line depicting the duration mice took to reach the target platform during training; (**F**) swimming paths of mice targeting the hidden platform during the final exploration trial. Data are presented as mean ± SEM (*n* = 6). * *p* < 0.05 vs. sham + Veh group; ^#^ *p* < 0.05 vs. AD + Veh group, ns: not significant.

To deepen our understanding of CBD’s therapeutic role in AD, we conducted in vivo studies in mice. Following the administration of aggregated Aβ_1–42_ into the lateral ventricle, a 12-day CBD (25 mg/kg) treatment regimen was initiated. The Morris water maze (MWM) test was utilized to evaluate the cognitive functions of the mice (Figure 2A). To ensure the absence of movement-related biases, we monitored the average swimming speed and distance on the inaugural day of the test (Figure 2B,C). Our data confirmed consistent mobility across all subjects. However, Aβ_1–42_-induced mice demonstrated extended platform localization times compared to the controls, highlighting Aβ_1–42_-induced cognitive impairments. CBD treatment significantly reduced escape latency, indicating its cognitive enhancement capabilities (Figure 2D). A comparison over a 5-day latency period revealed Aβ_1–42_′s detrimental impact on learning, which was partially mitigated by CBD (Figure 2E). Analyzing the mice’s navigational patterns in the maze further showcased CBD’s potential to improve spatial memory in AD models (Figure 2F). Collectively, our data support CBD’s promise in terms of addressing learning and memory challenges in AD mice.

### 3.3. Synaptic and Neurotrophic Modulation by CBD in Aβ_1–42_-Induced Mice

**Figure 3:**
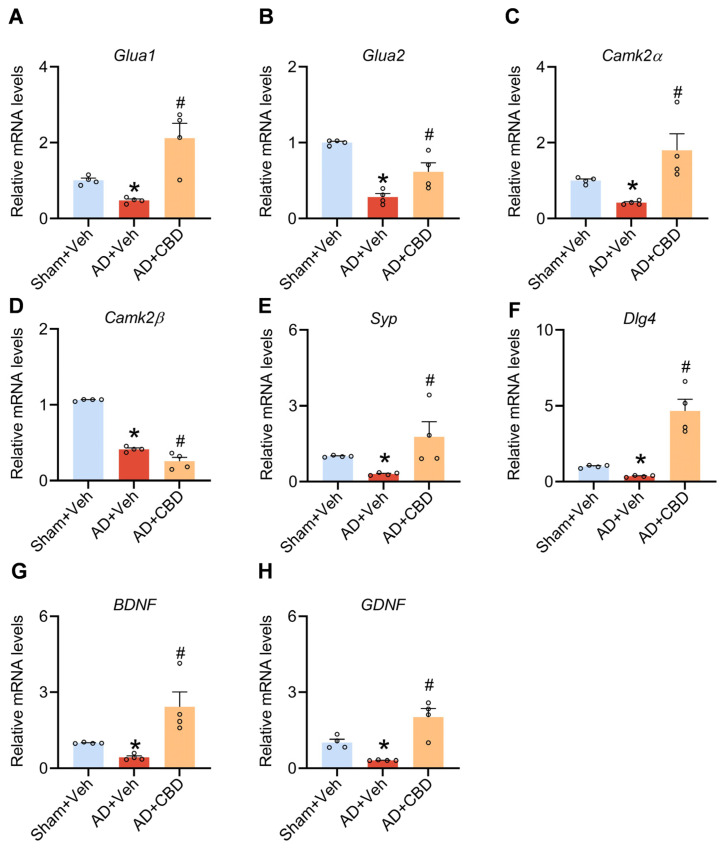
CBD’s neuroprotective role and synaptic dysfunction alleviation in Aβ_1–42_-induced mice. (**A**) Quantified image showing the mRNA expression level of Glua1. (**B**) Quantified image showing the mRNA expression level of Glua2. (**C**) Quantified image showing the mRNA expression level of CamKIIα. (**D**) Quantified image showing the mRNA expression level of CamKIIβ. (**E**) Quantified image showing the mRNA expression level of Syp. (**F**) Quantified image showing the mRNA expression level of Dlg4. (**G**) Quantified image showing the mRNA expression level of BDNF. (**H**) Quantified image showing the mRNA expression level of GDNF. Data are shown as mean ± SEM (*n* = 4). * *p* < 0.05 vs. sham + Veh group; ^#^ *p* < 0.05 vs. AD + Veh group.

Synaptic impairment is a hallmark of AD, profoundly affecting memory and cognitive function. Current therapeutic strategies targeting amyloid accumulation have not yet achieved the expected effects [30]. Consequently, the focus on rejuvenating neuronal circuits compromised by synaptic damage has gained traction as a reasonable route by which AD-associated memory abnormalities can be handled [31,32]. CBD’s established neuroprotective effects in vitro, as well as its potential to repair cognitive deficits in AD mice, warrant further exploration, particularly its influence on synaptic enhancement and neuronal protection in vivo. We analyzed the mRNA expression of the following essential synaptic proteins: GluA1 and GluA2 (AMPA receptor subunits); CaM-dependent protein kinase IIα (CamKIIα); synaptophysin (SYP); and DLG4, which encodes post-synaptic density protein 95 (PSD95) [33,34,35,36]. Our data indicate that CBD counteracts the Aβ_1–42_-induced decline in these mRNA levels in the mouse cerebral cortex (Figure 3A–E). However, no recovery of Camk2β levels was observed following CBD treatment (Figure 3F), suggesting that CBD may possess specific synaptic repair effects.

Neurotrophic factors, glial-cell-derived neurotrophic factor (GDNF), and brain-derived neurotrophic factor (BDNF) are pivotal for neuronal survival and regeneration [37]. In Aβ_1–42_-induced mouse models, their expression was significantly reduced, but CBD treatment markedly elevated their levels (Figure 3G,H). In essence, CBD appears to alleviate synaptic dysfunction and bestow neuroprotection in Aβ_1–42_-induced mice.

### 3.4. CBD’s Attenuation of Neuroinflammation and Glial Reactivity in Aβ_1–42_-Induced Mice

**Figure 4:**
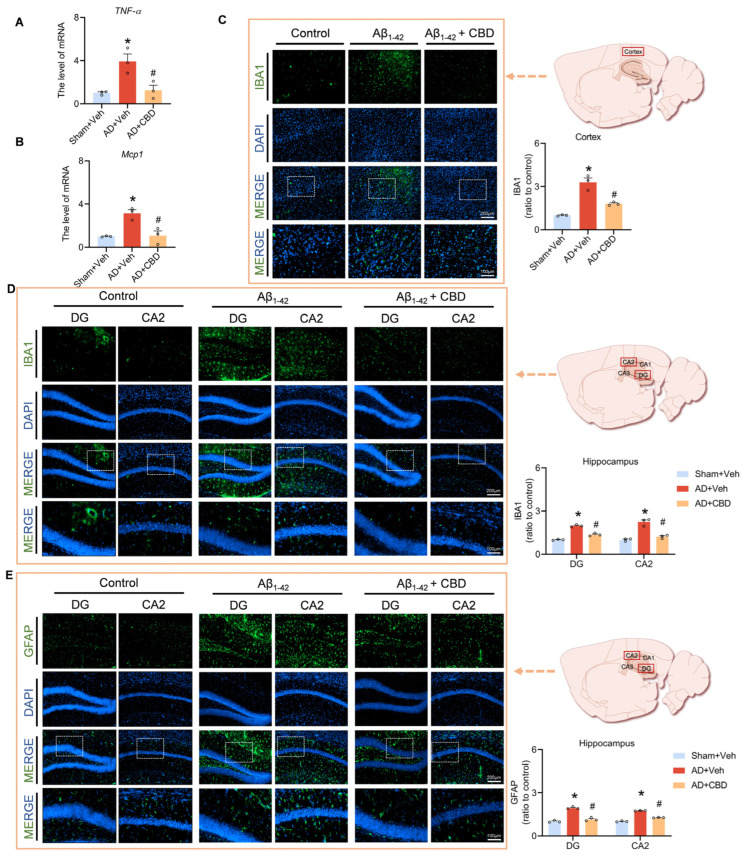
CBD’s modulation of inflammatory responses in Aβ_1–42_-induced mice. (**A**) Quantified image showing the mRNA expression level of TNF-α. (**B**) Quantified image showing the mRNA expression level of MCP1. (**C**) Representative fluorescence micrographs showing IBA1 expression in the cortex (scale bar: 200 μm) and quantification of the total number of IBA1^+^ cells in the cortex. (**D**) Representative fluorescence micrographs showing IBA1 expression in the DG and CA2 regions of the hippocampus (scale bar: 200 μm) and quantification of the total number of IBA1^+^ cells. (**E**) Representative fluorescence micrographs showing GFAP expression in the DG and CA2 regions of the hippocampus (scale bar: 200 μm) and quantification of the total number of GFAP^+^ cells. Data are shown as mean ± SEM (*n* = 3). * *p* < 0.05 vs. sham + Veh group; ^#^ *p* < 0.05 vs. AD + Veh group.

Research has highlighted the pronounced aggregation of microglia and astrocytes around amyloid plaques in AD brains, emphasizing their role in plaque phagocytosis and clearance. However, these plaques can induce a reactive response in microglia and astrocytes, leading to an inflammatory cascade that compromises synapses, induces neuronal apoptosis, and impairs memory. We analyzed the mRNA expression of critical proinflammatory markers to evaluate CBD’s anti-inflammatory potential in Aβ_1–42_-treated mice. Our data demonstrated that CBD significantly reduced mRNA levels of inflammatory agents, such as TNF-α and MCP-1, compared to Aβ_1–42_-treated mice (Figure 4A,B). Immunofluorescence studies using Iba1 have pinpointed heightened microglial activation in critical hippocampal regions, including the DG and CA2, as well as cortical areas, following Aβ_1–42_ exposure (Figure 4C,D). CBD treatment mitigated this activation.

Using GFAP to identify reactive astrocytes, we observed that CBD diminished their activation in Aβ-stimulated hippocampal regions (Figure 4E). Our findings suggest CBD’s efficacy in moderating microglial and astrocytic activation, offering anti-inflammatory benefits that protect synaptic function and alleviate AD-associated cognitive deficits. Our data support CBD’s potential therapeutic role in countering AD-related neuroinflammation.

### 3.5. CBD’s Modulation of Inflammatory Pathways and Synaptic Restoration in AD Mice

**Figure 5:**
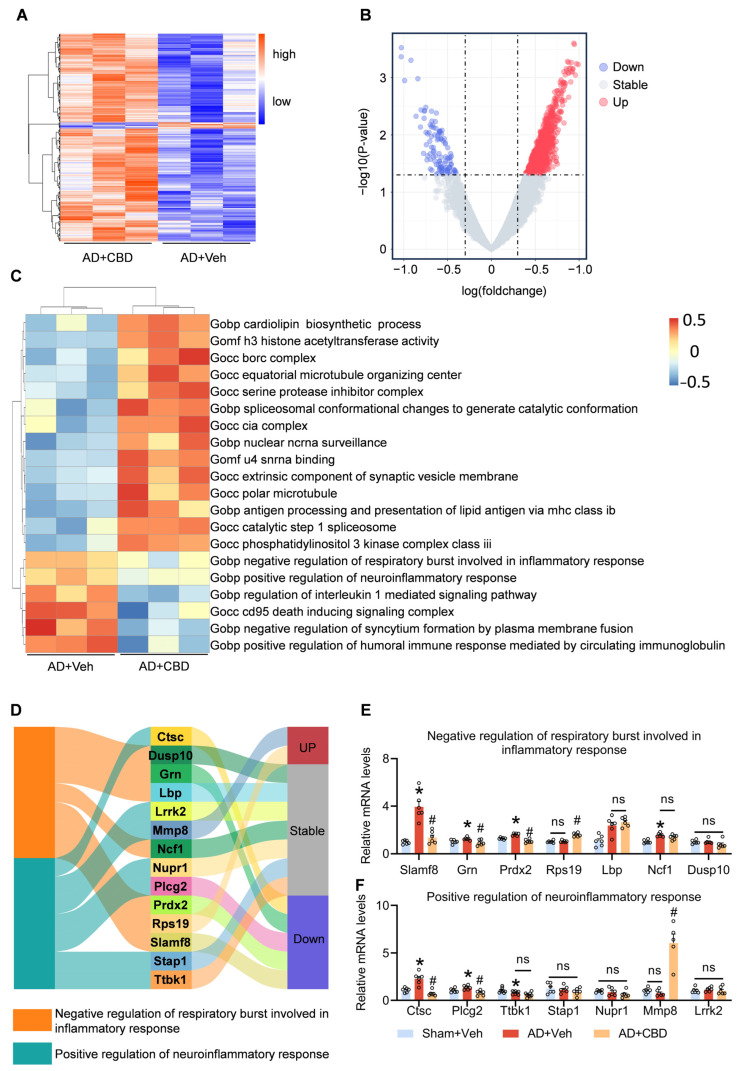
Revealing the underlying mechanisms of CBD’s anti-inflammatory effects. (**A**) Heatmap of DGEs in CBD-treated Aβ_1–42_-induced mice and Aβ_1–42_-induced mice. (**B**) Volcano plot of differentially expressed GO pathways in CBD-treated Aβ_1–42_-induced mice compared to Aβ_1–42_-induced mice. (**C**) Heatmap of the result of GSVA in CBD-treated Aβ_1–42_-induced mice and Aβ_1–42_-induced mice. (**D**) Sankey image showing the result of the gene expression related to the inflammation pathways. (**E**) Quantified image showing the mRNA expression levels of genes in the negative regulation of respiratory bursts involved in the inflammatory response pathway. (**F**) Quantified image showing the mRNA expression level of genes in the positive regulation of the neuroinflammatory response pathway. Data are shown as mean ± SEM (*n* = 3). * *p* < 0.05 vs. sham+Veh group; ^#^ *p* < 0.05 vs. AD+Veh group, ns: not significant.

We used gene set enrichment analysis (GSEA) of gene ontology (GO) on RNA-seq data from CBD-treated mice to decipher the molecular mechanisms driving CBD’s therapeutic efficacy, identifying differential pathways through Limma. Based on the *p* < 0.01, 3458 genes (Figure 5A) and 1051 differential pathways (Figure 5B) were found. Our analysis highlighted CBD’s potential to modulate inflammatory responses, particularly the positive regulation of neuroinflammatory responses (Figure 5C). RT-PCR analyses further revealed a down-regulation of the Slamf8, Grn, and Prdx2 genes, which are integral to the inflammatory response pathway, upon CBD administration (Figure 5D,E). Specifically, the signaling lymphocytic activation molecule family member 8 (Slamf8) amplifies inflammatory responses by increasing Toll-like receptor 4 (TLR4) expression in macrophages [38]. Progranulin (PGRN), encoded by Grn, modulates the MAPK and Akt pathways, with Pgrn deficiency potentially attenuating specific AD manifestations [39]. Peroxiredoxin 2 (Prdx2), a prevalent antioxidant enzyme in mammalian brains, plays a role in post-intracerebral inflammation [40].

Furthermore, CBD’s modulatory effects showered a decline in PLCG2 and CTSC expression, both implicated in the positive regulation of the neuroinflammatory response pathway (Figure 5D,F). Plcg2, or phospholipase c-γ2, is associated with microglia-driven innate immunity in AD, acting as a critical signaling hub for TREM2 functionality and microglial inflammatory responses [41]. Plcg2-KO has been shown to beneficially curtail proinflammatory cytokine secretion by microglia [42]. Cathepsin C (Ctsc) accentuates microglial M1 polarization and exacerbates neuroinflammation via the Ca^2+^-dependent PKC/p38MAPK/NF-κB pathway [43]. Its expression and activity are heightened in microglial neuroinflammation induced by lipopolysaccharides [44]. CBD down-regulates the expression of Ctsc, Grn, Slamf8, Plcg2, and Prdx2, predominantly curtailing microglial hyperactivity and associated inflammatory pathways.

**Figure 6:**
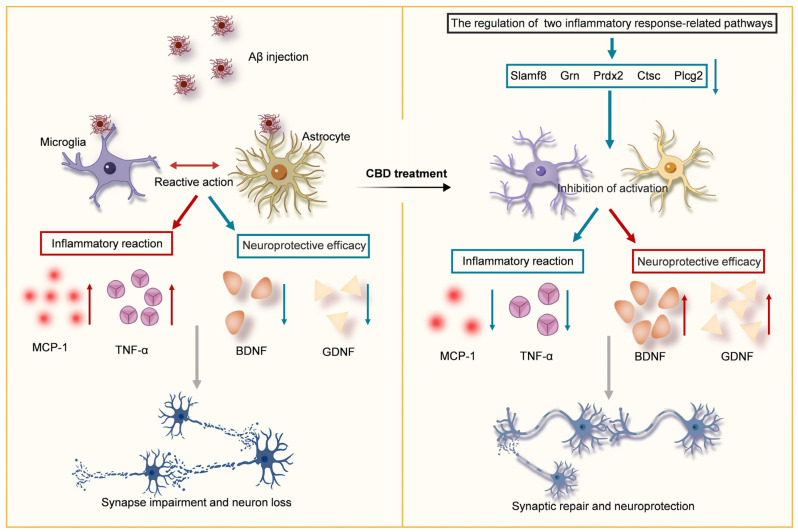
Schematic overview of CBD’s neuroprotective mechanism in Aβ-induced impairments. CBD can potentially modulate neuroinflammatory pathways, thus inhibiting inflammatory factor release and reducing synaptic damage. Concurrently, CBD augments neurotrophic factor expression, ensuring neuronal protection and ameliorating cognitive and memory deficits in AD mice. Downward arrows indicate negative regulation and upward arrows indicate positive regulation.

In summation, our data advocate for CBD’s potential to attenuate cognitive and memory deficits by modulating inflammatory and neuroinflammatory pathways. This dual action, encompassing synaptic restoration and neuroinflammation inhibition, bolsters cognitive function and mitigates memory challenges in AD mice (Figure 6).

## 4. Discussion

This study used in vitro methodologies to validate CBD’s safety profile and neuroprotective attributes. In vivo experiments further showcased CBD’s capacity to repair cognitive deficits and rejuvenate the learning and memory capabilities which had been compromised by Aβ in mice. Our analyses illuminated CBD’s ability to mitigate synaptic damage, neuronal attrition, and the hyperactivation of microglia and astrocytes while concurrently decreasing neuroinflammatory markers. RNA-seq data further underscored CBD’s anti-inflammatory prowess in terms of enhancing neuronal protection and memory function.

Synaptic plasticity is intrinsically tied to cognitive faculties, particularly learning and memory. Early AD manifestations include cognitive deficits, aberrant synaptic AMPAR distribution, and perturbed LTP/LTD, which are pivotal cellular mechanisms underpinning memory and learning. Aβ disrupts CaMKII activity, impairing AMPAR trafficking and, subsequently, LTP/LTD [45,46]. Our findings suggest that CBD has the potential to balance these Aβ-induced disruptions, particularly in the expression of CAMA, GLUA1, GLUA2, and PSD95.

Neuronal loss, which disrupts neural signaling circuits, is intimately linked to memory impairment. BDNF, with its broad neuroprotective effects in AD models, can reverse synaptic loss, restore cellular signaling, and prevent neuronal death [47]. GDNF overexpression in 3xTg-AD mice has shown cognitive improvements with concomitant BDNF up-regulation, suggesting a synergistic neuroprotective effect against neuronal atrophy [48,49,50].

In AD brains, Aβ polymers activate microglia and astrocytes, leading to an excessive release of proinflammatory agents and causing synaptic damage and memory impairment. Our data indicate that CBD suppresses this glial activation and reduces proinflammatory cytokine levels, notably TNF-alpha and MCP1. Sequencing data have further revealed CBD’s modulation of key inflammatory regulatory pathways and genes, including Plcg2, Ctsc, Slamf8, Prdx2, and Grn.

Chronic systemic inflammation may exacerbate the deposition of Aβ, increasing the risk of significant cognitive decline [51]. Immune components, as identified through genome-wide association studies, biomarker development, and animal model experiments, modulate the pathology of AD brains. Microglia, i.e., CNS-resident immune cells, are emerging as promising targets for disease-modifying strategies [52]. Our findings emphasize CBD’s potential in inhibiting microglial activation and reducing proinflammatory cytokine expression, positioning CBD as a promising therapeutic candidate for AD.

Nonetheless, there are inherent limitations involved in considering CBD as a therapeutic molecule for AD. A deeper understanding of CBD’s modulation of glial activation and its comprehensive effects on synaptic repair and neuronal protection is imperative. However, there is a paucity of research on glial cells, synapses, and neurons in relation to CBD, and this knowledge gap is crucial for the progression of CBD-based AD treatments [53]. Our results highlight CBD’s potential in ameliorating cognitive deficits in AD, primarily through synaptic restoration, neuronal protection, and the inhibition of glial activation and inflammatory factor release.

## 5. Conclusions

Our study has meticulously dissected the multifaceted neuroprotective and anti-inflammatory properties of CBD. We have demonstrated its capacity to counteract Aβ-induced cognitive and memory impairments, inhibit the hyperactivation of microglia and astrocytes, and augment the release of neurotrophic factors. Furthermore, our RNA-seq analyses have provided invaluable insights into CBD’s role in modulating critical genes within the inflammatory cascade, underscoring its robust anti-inflammatory potential.

Currently, cannabidiol (CBD), which is recognized for its non-addictive and non-toxic properties, is esteemed as a nutraceutical, offering therapeutic benefits across a spectrum of ailments, from ameliorating insomnia and alleviating depressive symptoms to optimizing lipid profiles and enhancing cardiovascular metabolism [54,55,56,57]. The equivalent dose for humans is equivalent to about 115 mg of CBD, which is sufficient for a 60 kg person, making this a viable oral supplement [58]. However, its potential role in AD prevention and management remains conspicuously absent from contemporary guidelines. Our research substantiates CBD’s efficacy in either preventing or mitigating the effects of AD. Thus, future formulations of CBD supplements might be strategically positioned to include indications for AD prevention and alleviation, expanding its therapeutic repertoire.

## Figures and Tables

**Table 1:** The primer sequences for RT-PCR are listed.

Gene	Primer Forward (3′ → 5′)	Primer Reverse (3′ → 5′)
*Bdnf*	TCATACTTCGGTTGCATGAAGG	AGACCTCTCGAACCTGCCC
*Camk2α*	ACCTGCACCCGATTCACAG	TGGCAGCATACTCCTGACCA
*Camk2β*	TCACCGACGAGTACCAGCTA	GGCAGATCCGAGCTTCTCTC
*Ctsc*	CCAACTGCACCTATCTTGACC	AAGGCAAACCACTTGTAGTCATT
*Dlg4*	TGAGATCAGTCATAGCAGCTACT	CTTCCTCCCCTAGCAGGTCC
*Dusp10*	CCATCTCCTTTAGACGACAGGG	GCTACCACTACCTGGGCTG
*Gapdh*	TGGCCTTCCGTGTTCCTAC	GAGTTGCTGTTGAAGTCGCA
*Gdnf*	TCCAACTGGGGGTCTACGG	GCCACGACATCCCATAACTTCAT
*Glua1*	TCCCCAACAATATCCAGATAGGG	AAGCCGCATGTTCCTGTGATT
*Glua2*	TTCTCCTGTTTTATGGGGACTGA	CTACCCGAAATGCACTGTATTCT
*Grn*	ATGTGGGTCCTGATGAGCTG	GCTCGTTATTCTAGGCCATGTG
*Lbp*	GATCACCGACAAGGGCCTG	GGCTATGAAACTCGTACTGCC
*Lrrk2*	GATCTCTGCACTCAGCTGTTTA	GCTTCTCACTGTCTTCCTCTTC
*Mcp-1*	GCATCCACGTGTTGGCTC	CTCCAGCCTACTCATTGGGATCA
*Mmp8*	CCAAGGAGTGTCCAAGCCAT	CCTGCAGGAAAACTGCATCG
*Ncf1*	ACACCTTCATTCGCCATATTGC	TCGGTGAATTTTCTGTAGACCAC
*Nupr1*	TCAACAGATGTCGGGGGAGA	TCTGCAGTGTGGGGCTTATG
*Plcg2*	CCGACTCTTACGCCATCA	GGGTAGCGAAGCCTCATC
*Prdx2*	GGTAACGCGCAAATCGGAAAG	TCCAGTGGGTAGAAAAAGAGGT
*Rps19*	CAGCAGGAGTTCGTCAGAGC	CACCCATTCGGGGACTTTCA
*Slamf8*	TCTCCTTCCCGTTGTGGTTG	CCAGATAGCCTCACGCACTTG
*Stap1*	CGGTCAGGATACCGGGAGTA	GCTCAGTAAGGCATGTGAGGT
*Syp*	GAGAGAACAACAAAGGGCCAA	GCGGATGAGCTAACTAGCCAC
*Tnf-α*	CCCTCACACTCAGATCATCTTCT	GCTACGACGTGGGCTACAG
*Ttbk1*	ATCCTGGAGTCCATTGAAGC	GCTAGCCCAAAGTCCAACAT

## Data Availability

The original contributions presented in the study are included in the article. Further inquiries can be directed to the corresponding authors.
